# Endocardial Pacemaker Implantation in Neonates and Infants

**Published:** 2006-04-01

**Authors:** Canan Ayabakan, Eric Rosenthal

**Affiliations:** Evelina Children’s Hospital, Guy’s & St Thomas’ Hospital Trust, Lambeth Palace Road, London SE1 7EH, UK

**Keywords:** Pacemaker, Endocardial pacing, Epicardial pacing, Lead extraction

## Abstract

Transvenous pacemaker lead implantation is the preferred method of pacing in adult patients. Lead performance and longevity are superior and the implantation approach can be performed under local anaesthetic with a very low morbidity. In children, and especially in neonates and infants, the epicardial route was traditionally chosen until the advent of smaller generators and lead implantation techniques that allowed growth of the child without lead displacement. Endocardial implantation is not universally accepted, however, as there is an incidence of venous occlusion of the smaller veins of neonates and infants with concerns for loss of venous access in the future. Growing experience with lower profile leads, however, reveals that endocardial pacing too can be performed with low morbidity and good long-term results in neonates and infants.

Cardiac pacing in infants and children has evolved considerably since the initial implants over half a century ago. Pacemaker generators have become smaller and are now easily accommodated subcutaneously or submuscularly either in the chest or the abdominal wall in the smallest of infants. Improvements in lead technology have enabled lower chronic thresholds with greater mechanical and electrical integrity and longevity of the leads and the pacemaker system. While endocardial leads have consistently proven to be superior to epicardial leads both mechanically and electrically, the development of steroid eluting epicardial leads has improved the effectiveness of epicardial systems [1]. The child who receives a pacemaker will undergo several additional interventions to replace the generator with or without the lead itself during his/her lifetime. Special consideration should therefore be given when selecting the most appropriate pacing system in the very small.

The choice of the method of permanent pacing is determined by the patient’s size, and presence of structural heart disease. While a large body of opinion holds that neonates and infants should receive epicardial pacing systems [2-4], experience with endocardial leads in the smallest of infants is building up (Figure 1). The favourable outcome of endocardial leads in small infants is not limited to anecdotal case reports [5-10]. The largest experience to date from two centres (Guy’s Hospital and Wilhelmina Children’s Hospital) in 39 infants ≤ 10kg was published recently by Kammeraad et al [11]. After a relatively long follow-up period of a median 4.3 years (up to 15.3 years), 31 of 36 patients (86%) continued with an endocardial system and 27 of them had the original endocardial lead. Two endocardial systems had to be replaced with epicardial systems and pacing therapy was stopped in three patients because it was no longer indicated. Lead survival was excellent with 87% of those with their first or second generator continuing with the original lead - comparable to the results of epicardial leads in older children [12]. Indeed in a similarly aged cohort, a significant number of epicardial lead failures were reported in neonates within the first year of implantation due to acute exit block and lead fractures [13].

## Venous obstruction

Endocardial leads are increasingly preferred for small infants in other centres as well [14-16]. The reasons for this preference are lower acute and chronic threshold values and decreased surgical morbidity from the procedure. The risk of venous occlusion, however, is the major drawback of endocardial lead placement in neonates and infants. If an adequate amount of slack is not left in the heart to cater for growth and the lead cannot be advanced, then replacement will be needed. If the lead cannot be replaced due to venous occlusion then the contralateral subclavian vein will need to be used and this may reduce the options for venous access in the future. Although transfemoral and transhepatic pacing can be performed with good results, few centres advocate these routinely [17,18]. Accordingly some centres still prefer to implant epicardial leads in children less than 10 kg [2-4,13]. In some centres the policy is one of replacing epicardial systems electively with an endocardial system at the end of life of the first generator due to an increase in lead fractures with time [13].

Studies specifically comparing pacing via the endocardial route versus the epicardial route in small infants are lacking. The incidence of venous obstruction and occlusion has rarely been addressed. Venous obstruction due to endocardial leads in a small group of newborns and infants was assessed by Stojanov et al [16]. In 12 children younger than 12 months, who had endocardial leads implanted via the cephalic vein, only two had ultrasonographic evidence of partial (up to 20%) venous occlusion at the end of a mean follow-up of 85.2 pacing months. Only four patients were reported to have asymptomatic venous occlusion during lead extraction among the 36 neonates and infants reported by Kammeraad et al [11]. Asymptomatic venous occlusion was not systematically looked for however, and the incidence may therefore be higher in this cohort who had their leads implanted via a subclavian puncture. Even in older children, the incidence of venous occlusion may be as high as 21% in endocardial leads depending on the lead diameter, body surface area and the introduction site (subclavian vein versus cephalic vein) of the lead [19].

Stojanov et al promote a cephalic vein cut down rather than a subclavian puncture approach in the very small [15]. It may be that avoidance of the relatively large sheath needed for a subclavian puncture to introduce the lead tip, which is larger than the lead body, has an advantage. Whether late venous obstruction depends more on the size of the subclavian introducer and acute trauma produced at the time of vein puncture or the diameter of the lead left in the vein has not been systematically investigated.

## Lead Extraction

**Figure 2:**
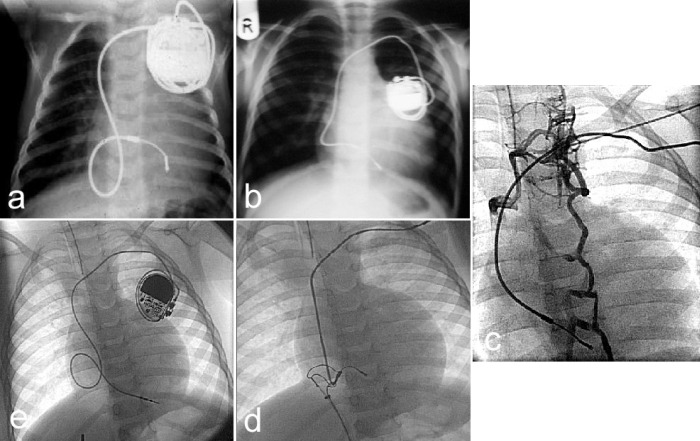
An infant with congenitally corrected transposition (cTGA) developed heart failure with the onset of complete heart block and was paced at 6 weeks of age (**a**). At 4 years the generator reached its end of life with some remaining lead slack (**b**). During the generator change, the lead was inadvertently damaged and had to be replaced. Angiography revealed an occluded subclavian vein (**c**). To avoid the use of large countertraction sheaths from the subclavian approach, a coronary guidewire was passed into the lead stylet channel and was drawn out the femoral sheath with the lead after snaring the lead with the help of a tip deflector wire (**d**). Over the subclavian-femoral guidewire circuit, a new sheath was placed to implant a new ventricular lead (**e**).

Endocardial pacing in neonates is not for the faint-hearted as lead replacement is likely to be needed in at least some of the patients. While extracting 11 leads uneventfully in nine patients (including atrial leads placed at the second or third system), Kammeraad et al [11] had to abandon a ventricular lead, which was adherent to the atrial myocardium. Extraction techniques are continuing to improve and lead replacement through an occluded subclavian vein is now possible using countertraction sheaths. A technique for femoral extraction that allows preservation of guidewire access across an occluded subclavian vein (Figure 2) is described by Kammeraad et al [11]. Downsizing extraction countertraction sheaths for smaller leads may allow their safe use even in small children. In older children, standard diathermy and laser extraction sheaths can be used safely.

## Concomittant Cardiac Surgery

Although epicardial leads do not carry a risk of venous thrombosis, there is an increased perioperative morbidity [13]. Patients who are already a candidate for a sternotomy or thoracotomy for the correction of heart defects may benefit from epicardial leads especially when dual chamber pacing is required for atrioventricular synchrony to improve the postoperative hemodynamics. However postoperative infections and pericarditis may preclude epicardial lead placement, in which case an endocardial pacing system is the best choice [11,13]. While some surgical heart block patients require dual chamber pacing, the majority of infants and neonates who require pacing, are well served by a single chamber system that can be implanted endocardially. Transvenous dual chamber pacing is possible but generally avoided due to the increased risks of venous occlusion [11,14]. Early pacing with a dual chamber system may lead to a cardiomyopathy due to pacing induced ventricular dysynchrony which is aggravated by the higher rates found with atrial tracking in the very young [20,21]

## Conclusions

Neither the epicardial nor the endocardial approach in permanent pacing is free from complications. The experience reported with endocardial pacing in neonates and infants while encouraging is limited and no directly comparative data with epicardial pacing are available. Although the clear advantage of endocardial pacemaker implantation over the epicardial approach has yet to be demonstrated, the growing experience indicates that endocardial pacing is feasible and effective even in neonates and small infants - and it is an acceptable alternative to epicardial systems. The current disadvantages of endocardial leads may be overcome in the future by downsizing of the leads and extraction systems. The ultimate approach in very small children currently will depend on the facilities and experience of the surgeons and cardiologists in each centre.

## Figures and Tables

**Figure 1:**
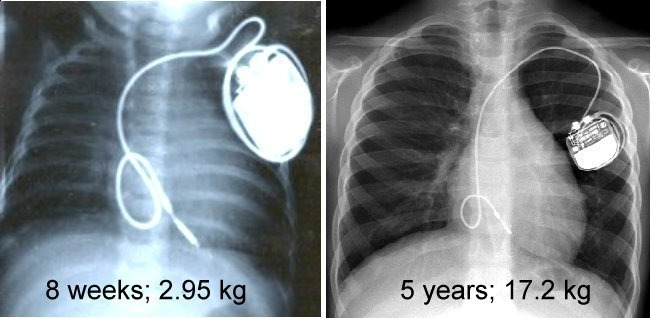
Left panel shows lead position in a baby with congenital complete heart block who was born prematurely with hydrops. A transvenous endocardial pacemaker was implanted at the age of 8 weeks when she weighed 2.95 kg. At 5 years of age (weight 17.2 kg), she still has the initial lead and generator (right panel). The redundant loop of lead formed in the right atrium has not yet been taken up and will allow generator replacement without the need for lead advancemenrt.
